# Sustainable and acceptable school meals through optimization analysis: an intervention study

**DOI:** 10.1186/s12937-020-00579-z

**Published:** 2020-06-24

**Authors:** Patricia Eustachio Colombo, Emma Patterson, Anna Karin Lindroos, Alexandr Parlesak, Liselotte Schäfer Elinder

**Affiliations:** 1 Department of Global Public Health, Karolinska Institutet, 171 77 Stockholm, Sweden; 2 Centre for Epidemiology and Social Medicine, Region Stockholm, 113 65 Stockholm, Sweden; 3 The Swedish Food Agency, 753 19 Uppsala, Sweden; 4 Department of Internal Medicine and Clinical Nutrition, the Sahlgrenska Academy, University of Gothenburg, 405 30 Gothenburg, Sweden; 5 Global Nutrition and Health, University College Copenhagen, 2200 Copenhagen, Denmark

**Keywords:** Children, Public meals, Sustainable diets, Agenda 2030

## Abstract

**Background:**

School meals hold considerable potential to shape children’s diets and reduce food-related greenhouse gas emissions (GHGE)—in the short and long term. This study applied linear optimization to develop a GHGE-reduced, nutritionally adequate, and affordable school lunch menu. The effects on food waste, consumption and pupils’ satisfaction with the meals were evaluated.

**Methods:**

A pre-post design was employed to assess the effects of implementing an optimized lunch menu on daily food waste, consumption, and pupils’ school meal satisfaction in three schools (grades 0–9) from one Swedish municipality. A food list containing amounts, prices, nutrient content, and GHGE-values of all foods used for a previously served (baseline) four-week lunch menu was created. Using linear programming, this food list was optimized for minimum deviation and constrained to ensure nutritional adequacy and a reduced climate impact. The optimized food list was developed into a new (intervention) four-week lunch menu by a professional meal planner, following the baseline menu as closely as possible. The baseline and intervention menus were served for four weeks, respectively, with a two week break in between. Prepared, wasted and leftover food were weighed daily by the school kitchen staff during both periods. Interrupted time series analysis assessed mean and slope differences in daily food waste and consumption between the two periods. School lunch satisfaction was assessed with an online questionnaire at baseline and during the intervention.

**Results:**

Optimization resulted in a food list that was 40% lower in GHGE, met all nutrient recommendations for school meals, and cost 11% less compared to baseline. The intervention menu was served as planned, with only minor changes required (for practical reasons). Plate waste, serving waste, consumption and school lunch satisfaction did not differ significantly from baseline, in any of the schools.

**Conclusions:**

The findings demonstrate that school meals can successfully be improved regarding health and environmental sustainability using linear optimization, without negative effects on food waste, consumption or cost. This approach offers the necessary flexibility to tailor menus towards different priorities and could therefore be transferred to other types of meal services.

**Trial registration:**

The trial is registered at clinicaltrials.gov (NCT04168632 Fostering Healthy and Sustainable Diets Through School Meals (OPTIMAT).

## Background

The environmental impact of our food system is substantial [1]. According to the 2019 report from the Intergovernmental Panel on Climate Change, 25–30% of total greenhouse gas emissions (GHGE) are attributable to the food system through agriculture and land use, storage, transport, packaging, processing, retail, and consumption [2]. In addition, unhealthy diets contribute significantly to the global burden of disease by increasing the risk of cardiovascular diseases, cancers and type-2 diabetes [3]. The 2019 EAT-Lancet report [4] recommends a global transformation of food systems to improve health, safeguard environmental stability and thus ensure the attainment of Agenda 2030 for Sustainable Development [5], and the Paris Agreement requiring that the increase of the planet’s temperature stays below 1.5 °C [6]. Central to this transformation is the wide-scale adoption of sustainable diets that are “*protective and respectful of biodiversity and ecosystems, culturally acceptable, accessible, economically fair and affordable; nutritionally adequate, safe and healthy; while optimising natural and human resources*” [7].

One area with the potential to change demand and promote sustainable eating is through more sustainable procurement of food in the public sector [8]. In particular, school meals have been highlighted as a way for children to adopt healthy and sustainable dietary patterns [4, 9] which may persist throughout life. Planning of school meals need to take many aspects into account: nutritional needs must be met, environmental impact must be minimized, meals must be acceptable to pupils and affordable to providers. Being able to succeed in considering all these aspects simultaneously requires that the professionals producing these meals possess the necessary knowledge, skills and tools.

Roughly 1.1 million fully subsidized school meals are provided daily to all children aged 6 to 15 years in Swedish primary schools [10]. Lunches are typically self-served, hot, main dishes including a salad buffet, bread, spread and milk or water. These lunches should be nutritious and cover 30% of children’s dietary needs over approximately four weeks [11]. School lunches contribute substantially to the nutritional quality of Swedish children’s diets, providing on average almost half of total daily vegetable intake, two-thirds of daily fish intake, and one third of daily meat intake [12]. Each year, approximately 196 million meals, at a cost of around 640 million Euro (EUR), are served in Swedish primary schools [10]. Due to their reach and scale, school meals have great potential to increase children’s knowledge about health and sustainability, foster sustainable dietary habits, as well as to increase the amount of food purchased through sustainable public procurement. Currently, many schools in Sweden are making changes to their meal plans in order to make meals more healthy and sustainable, and they continuously monitor the meal quality [13], yet tools to systematically reduce the environmental impact of school meals while ensuring nutritional requirements, acceptability and affordability are still lacking.

Optimization is a suitable mathematical method for finding the best solution among several different, occasionally competing, demands. In the case of sustainable eating, such demands would be nutritional adequacy, environmental impact, (cultural) acceptability, and affordability [14, 15]. Optimization has previously been used to model diets that are cost-effective, nutritionally optimal, and more climate friendly [16–22]. The method has also been applied to model meals both in Spanish [23] and in Swedish school contexts [24]. However, to the best of our knowledge, no attempt has been made test the feasibility of combining the application of a holistic linear optimization method to the planning of school meals in practice.

This study aimed to: (i) apply linear programming to develop a GHGE-reduced, nutritionally adequate, and affordable four-week lunch menu optimized for minimum deviation from the current food supply (the latter as a means to maximize acceptability); and (ii) to evaluate the new menu’s effects on food waste, food consumption, and pupils’ satisfaction with school lunches. Our hypothesis is that school meals can be optimized to be nutritious and more climate friendly, without negatively affecting acceptance, food waste and cost.

## Materials and methods

### Study design

We employed a pre-post design with interrupted time series (ITS) analysis to assess the effect of implementing an optimized lunch menu on daily food waste and consumption at school level. ITS analysis is one of the strongest methods to evaluate population-level interventions that are implemented at a clearly defined point in time and when randomisation is not possible or not meaningful [25].

For a baseline period of four weeks, during the spring term of 2019, children in three primary schools received the usual (baseline) menu with two daily meals to choose from. After a two-week break (one week’s mid-term break plus an extra week required by the school meal manager), the optimized four-week menu was served daily for four weeks (intervention period).

### Theory

The intervention builds on Social Cognitive Theory (SCT), which states that behavior is determined by reciprocal interaction between personal factors and the social and physical environment [26]. Since this was the first time such an intervention was evaluated, we made the decision to target the physical environment only by introducing a new school lunch menu. A social (pedagogical) component targeting the pupils in the intervention was thus purposefully not included.

### Recruitment of schools

During the fall of 2018, public meal managers in three municipalities of Stockholm, Sweden, were invited to information meetings about the project. These stakeholders were chosen as they had previously demonstrated interest in sustainable school meals. The managers were asked to invite school head chefs who were interested in the issue of sustainability. Six primary schools (up to grade 9) attended the information meetings. To be eligible for inclusion in the intervention, the schools needed to have on-site kitchens, and be able to provide previously used recipes for a standard four-week menu electronically (critical for the optimization process). Three of the interested schools (with 627, 630, and 378 pupils in grades 0–9, respectively), from one of Stockholm county’s 26 municipalities met inclusion criteria and were thus eligible to participate in the intervention.

The proportion of students with a non-Swedish background was 23% (School 1), 80% (School 2) and 73% (School 3), with 50% being the average in the municipality. The share of pupils with parents without a post-secondary education was 43% (School 1), 72% (School 2) and 60% (School 3) with 51% as the average in the municipality as a whole. The municipality provides the same lunch menu for all of its schools. School chefs are expected to follow this menu but have some degree of freedom to adapt the menus to suit local preferences.

Additional meetings were held with the entire staff at each of the three schools to explain the purpose of the study, to present the timeline for the upcoming intervention and to spread awareness about the intervention throughout the school organization. Schools were asked to inform parents via their usual newsletter. Separate meetings were subsequently held with the school restaurant staff, to provide detailed instructions on how to perform the waste and consumption measurements. Practical help with performing the measurements during the intervention was offered but all schools declined. Written agreements with all participating schools concerning the intervention and tasks to be performed by the school and by the research team, respectively, were draw up between the headmaster and the principle investigator (LSE).

### Optimization

#### The baseline menu

The municipality’s menu is planned to meet the nutritional needs for a reference pupil aged 10–12 years over a four-week period. The municipality provided a four-week menu which had previously been served at the recruited schools (Additional file 1), together with the 40 recipes (2 dishes/day over a period of 20 weekdays). The recipes indicated the amounts of each food item used in kilograms (kg) of raw food as well as the corresponding price of each item (total cost and price per kg). The recipes also included foods used for the daily salad buffet. The total weight for each food item in the baseline menu was calculated, as was the cost, based on the average price paid per kg of that food item. After excluding spices, which were present in negligible amounts and contributed only marginally to the nutrient supply, the baseline menu was converted to a list of 147 food items together with corresponding amounts and prices. This food list was then linked to the Swedish Food Agency’s food database [27]. Data on the GHGE of the foods, expressed as carbon dioxide equivalents (CO_2_eq), was added by linking to the Climate database maintained by the Research Institutes of Sweden, RISE. This database builds on results from life cycle analyses [28, 29] and the characteristics of typical Swedish food purchasing patterns [30]. All calculations of nutritional adequacy are based on the nutrient content of the edible shares of prepared (cooked, simmered, fried, baked etc.) foods, while cost and CO_2_eq are based on the amount of raw food, as purchased. The edible proportions and yields of raw foods were calculated as described previously [15].

#### Optimization using linear programming

Linear programming (LP) is the application of an algorithm for maximizing or minimizing a given linear objective function (the variable to be optimized) subjected to a set of linear constraints (criteria to be met) on a list of decision variables (the variables that are changed by the model) [31]. The baseline food list was optimized following a strategy previously developed by us and described in detail elsewhere [24]. Briefly, the baseline menu provided an average of 674 kcal per lunch which is in line with current recommendations for a school lunch [11]. This value, together with the total amount of kcal for all food items purchased for the baseline menu, was used to calculate an energy-standardized food supply for one reference pupil and lunch. These energy-proportional shares of each food item for one pupil and lunch, representing the baseline food supply for the four-week period, were calculated and used in the optimization. As the objective function of the LP model, we chose the minimization of the total relative deviation (TRD) from the baseline food supply [24]. The purpose of this was to keep the optimized food amounts as similar as possible to the baseline values in order to make the new menu as acceptable as possible. At comparable GHGE reductions, using TRD as the objective function was previously shown to provide models with a higher degree of similarity to the baseline food supply compared to using GHGE as the objective function [24]. The decision variables were the edible amounts of foods that were eligible to be included into the optimized food supply. The constraints applied (Additional file 2) were the nutritional recommendations for a school lunch and reference pupil (11), and a level of GHGE (CO_2_eq) which was 40% lower than the baseline level, reflecting the goal of GHG-reductions set by the European Commission [32]. Additionally, individual food items were constrained to decrease by a maximum of 75% or increase by a maximum of 100% from baseline levels [24]. Consequently, no foods were removed entirely, and no new foods were added to the list. The average relative deviation (ARD) from the baseline food supply, calculated by dividing the TRD by the total number of food items included in the model, was used to estimate the similarity between the baseline and the optimized menu.

Milk (not used for cooking) and crisp bread, which are traditionally always available for consumption ad libitum at lunch, were included in the optimization but their amounts were not allowed to be changed by the algorithm. Without this exception, there was a risk that the amount of milk would be reduced, and that pupils arriving later for lunch would have less or no milk available to them. Linear optimization was performed with the CBC (Coin-or branch and cut) Solver algorithm, which is part of the Excel® 2016 software add-in OpenSolver, V. 2.8.6 [33].

In order to illustrate the changes occurring, foods were grouped into 11 categories, and the changes between and within food groups were described. The categories were those used in the Climate database [30]: Beverages (milk excluded); Fats and oils; Seafood; Fruits and berries; Vegetables and roots; Meat; Cereals; Dairy; Nuts and seeds; Seasoning and sauces; Sugar and sweets.

### Meal planning and implementation

The optimized food list was then provided to a professional meal planner from an unrelated municipality, with prior experience of planning meals with reduced climate impact for schools. The meal planner was also provided with the baseline menu and recipes so that the new menu could be developed following the baseline menu as closely as possible. The decision to try to resemble the baseline menu, rather than develop radically new dishes, was taken by us in consultation with the intervention municipality’s meal manager. Following an iterative process, where the meal planner was granted the possibility to make minor changes to the optimized food amounts (e.g. reducing amount of potatoes, reported in the Results section) in consultation with the research team, a new menu was developed (Additional file 1). As an initial verification of its practical applicability, the new menu was discussed with, and approved by, the municipality’s meal manager and the school chefs.

As mentioned previously under “Theory”, we decided to keep the information about the intervention to pupils and parents to a minimum, and no other intervention components such as pedagogical activities related to sustainable eating were included. This decision was taken since the aim of this first intervention study, with an uncontrolled design, was to evaluate the effect of the new menu on food waste, consumption, and school-meal satisfaction, and not the potential effects of other intervention components.

During the course of the intervention, the school chefs were granted some flexibility to suggest minor adjustments to the new menu, in cases where they judged that the meals were likely to be rejected by a significant number of pupils. The proposed changes were first discussed with the research team before being implemented. Examples of these changes are described in the Results section. The kitchen staff at each school prepared the baseline and new menus in the school kitchen and served the meals in the canteen as usual. The staff consisted of one head chef, one chef, and two to four kitchen assistants, depending on the school.

### Outcomes

#### Food waste and consumption

To assess potential unwanted effects of the new optimized menu, data on food waste and consumption were collected daily in each school during the four-week baseline period and during the four-week intervention period. No measurements were taken during the two-week break between periods.

A method for measuring food waste and consumption was used which combined the Swedish Food Agency’s method for measuring waste in kitchens producing public meals [34] with School Food Sweden’s method for measuring school lunch consumption [35].

The following components were weighed by school kitchen personnel (using school kitchen scales) and recorded on a form: all food waste generated during preparation and cooking such as peel, pits and bones (“kitchen waste”); all food prepared in the kitchen (“prepared food”); food that was prepared but not eaten by the pupils and had to be thrown away (“serving waste”); food that had not yet been brought out to the canteen and could be re-used (“leftover food”); and the food discarded by the pupils in a bin when leaving the school canteen (“plate waste”). The number of plates used by the pupils in the school restaurant was also recorded by the kitchen staff daily and was used to estimate the number of pupils eating the school lunch that day. Total daily food consumption (kg) was calculated by subtracting the weight of serving waste, plate waste, and leftover food, from the weight of the prepared food (Table 1). Plate-waste per pupil was calculated for each day by dividing the total amount of plate waste by the total number of plates used that day (Table 1). Similarly, the consumption per pupil was calculated for each day by dividing the total consumption by the total daily number of plates used that day (Table 1). Total plate waste, plate waste per pupil, serving waste, total consumption, and consumption per pupil were considered to be directly related to the pupils’ acceptability of the new menu.

**Table 1:** Calculated variables for food waste and consumption

Variable	Calculation
Total daily food consumption (kg)	Total daily prepared food - (total daily serving waste + total daily plate waste + total daily leftover food)
Plate waste/pupil (g)	Total daily plate waste/total daily number of plates
Consumption/pupil (g)	Total daily consumption/total daily number of plates

To keep track of how well the schools implemented the new menu and waste measurements, we maintained regular contact with schools. The head chefs provided the research team with weekly reports of all waste measurements and details of any potential adjustments that had been made to the new menu. Members of the research team called the head chefs at the end of each week to remind them about the weekly report, and also visited each school once during both baseline and intervention periods. Head chefs were given the opportunity to raise concerns with regards to the new menu or the waste measurements during both calls and visits. A meeting was also held with the three head chefs and the municipality’s meal manager in-between baseline and intervention periods to ensure the quality of the measurements in the weekly reports as well as to discuss the new menu. All correspondence with the school chefs was logged.

#### Satisfaction with school meals

Satisfaction with school meals among pupils in grade 5 and 8 was assessed twice with an online questionnaire. The first occasion took place in the middle of the baseline period and the second in the last week of the intervention period. The reason for choosing grade 5 and 8 was to enable a comparison with data from a recent national dietary survey (Riksmaten Adolescents) that also covered indicators/dimensions of school-meal satisfaction in these grades [36]. The questionnaire was answered anonymously at each school. The questionnaire is used regularly by a large number of schools through the online quality monitoring tool School Food Sweden [13] and a very similar questionnaire has been found to have good face validity [37]. It contains ten questions referring to the school lunch (Additional file 3) with a varying number of response options. Five of the ten questions specifically cover their usual (i.e. not for that specific day or week) satisfaction with the school lunch. These questions include: how often they consider that the school lunch tastes good (always/almost always/rarely/never), how many days per week they tend to eat school lunch (never or almost never/1 day a week/2 days a week/3 days a week/4 days a week/5 days a week), how often they usually feel full after having eaten the school lunch (always/almost always/rarely/never), and how often they tend to leave plate waste (always/almost always/rarely/never). They were also asked to provide an overall rating of the school lunch (very good/good/not so good/bad) (Additional file 3). The pupils could also answer “Don’t know” to this question, but none of the pupils selected this option. For the analyses, the five items were re-coded to obtain more equally sized groups (see details on how the answers were grouped in Table 4).

### Statistical analyses

The software R (version 3.6.1) [38] was used for statistical analysis. All tests were two-sided and *P* values of < 0.05 were assumed to indicate statistical significance. Summary statistics were performed at aggregated (school) level to produce means, 95% confidence intervals (CIs) and proportions (%).

In order to compare food waste and consumption between the two time sequences (baseline and intervention), interrupted time-series (ITS) analysis was used [25]. The following outcomes were compared between the two time periods: the number of pupils consuming lunch (n), kitchen waste (kg), prepared food (kg), serving waste (kg), total plate waste (kg), plate waste per pupil (g), total consumption (kg), and consumption per pupil (g).

The ITS analysis was based on a priori defined segmented linear regression models corresponding to the baseline and the intervention period [25]. All data points were included, and separate lines were fitted to each time-interval (baseline and intervention) for each outcome (Y). These models were determined by three independent variables: (i) a time variable, applied to adjust for a potential baseline trend; (ii) a variable indicating whether the data point represented the baseline or intervention period and thus included in the model to assess the level change (mean difference) resulting from the intervention; and (iii) an interaction term between (i) and (ii) specifying the slope change resulting from the intervention. A significant slope change indicates that there is a difference in how much the outcome (Y) changes with one unit increase in X (days) between baseline and intervention periods. Assumptions of normality, homoscedasticity, as well as absence of autocorrelation and partial autocorrelation were assessed through normal probability plots, residual plotting, and the Durbin-Watson test [25].

The effect of the new menu on the five questionnaire items related to school meal satisfaction was explored by means of Pearson’s chi-squared test (χ2) within each school. The data from the three schools was also combined and compared (baseline vs. intervention).

## Results

### Linear optimization and menu planning

Table 2 shows the GHGE, cost, ARD, and nutritional adequacy of the baseline food list, in the optimzied food list, in the new (planned) menu and in the served (delivered) menu. The baseline food list contained 829 g CO_2_eq per meal, cost 11.3 Swedish Krona (approx. 1.05 Euros). It met all dietary reference values used for planning school meals, except for saturated fatty acids (7% over target) and iron where the recommended intake (RI) was 10% under the target. The linear optimization resulted in a food list that had 40% lower GHGE, cost 11% less, and met all nutritional requirements. The ARD was 15.2% from baseline.

**Table 2:** Greenhouse gas emissions (CO_2_eq), cost, average relative deviation, and nutritional adequacy per meal in the baseline food list, optimized food list, planned (new) menu and served (delivered) menu in the three schools

Parameter		Baseline list	Optimized list	Planned menu	Delivered School 1	Delivered School 2	Delivered School 3
CO_2_eq, average per meal	g	829	497	496	482	501	498
	% change	na	−40.0	−40.2%	−41.9%	−39.6%	−39.9%
Cost, average per meal	SEK	11.3	10.01	9.76	10.50	10.01	9.76
	% change	na	−11%	−14%	−7%	− 11%	−14%
Average relative deviation (ARD) (ARD	%	na	15.2	16.2	16.9	19.7	16.4
Energy	%^Target^	100	100	97*	98*	98*	97*
Carbohydrates	%^Lower^	96	102	96	96	99	96
	%^Upper^	75	79	76	77	79	77
Fat	%^Lower^	148	139	137	140	137	137
	%^Upper^	96	90	91	93	90	91
Protein	%^Lower^	166	159	156	160	159	156
Fiber	%^Lower^	150	179	170	168	171	169
Saturated fatty acids	%^Upper^	107*	85	86	88	86	86
Monounsaturated fatty acids	%^Lower^	171	166	165	169	164	165
Poly unsaturated fatty acids	%^Lower^	147	147	146	147	145	146
Vitamin A	%^RI^	231	150	147	146	149	147
Vitamin D	%^RI^	149	148	145	144	145	145
Vitamin E	%^RI^	266	267	262	263	265	262
Thiamine	%^RI^	153	156	150	150	152	150
Riboflavin	%^RI^	123	113	110	113	112	110
Vitamin C	%^RI^	303	259	211	208	228	211
Niacin	%^RI^	135	133	117	119	130	117
Vitamin B6	%^RI^	178	175	155	156	165	155
Vitamin B12	%^RI^	238	192	189	193	191	189
Folate	%^RI^	257	282	268	267	268	268
Phosphor	%^RI^	279	268	261	265	266	260
Iodine	%^RI^	254	251	240	237	243	241
Iron	%^RI^	90*	100	97*	99*	97*	96*
Calcium	%^RI^	138	113	112	113	112	113
Potassium	%^RI^	130	131	118	118	122	117
Magnesium	%^RI^	138	149	141	141	143	140
Salt	%^Upper^	74	69	72	76	72	73
Selenium	%^RI^	108	100	99*	101	102	99*
Zink	%^RI^	106	100	98*	99*	98*	98*

*Deviation from energy target for a school lunch (30% of daily estimated energy requirement), from the recommended intake (RI) for a school lunch (30% of daily RIs), or from recommended lower or upper intake levels for a school (30% of daily recommended lower or upper intake level)

%^Target^, the relative coverage of the energy target for a school lunch

%^RI^, the relative coverage of the RIs for a school lunch

%^Lower^, the relative coverage of the recommended lower intake level for a school lunch

%^Upper^, the relative coverage of the recommended upper intake level for a school lunch

SEK = Swedish Krona

na = not applicable

A few adjustments to the optimized food list were made at the meal planning stage. The changes consisted of a reduction in potatoes (− 29%), the amount of which had doubled in the optimized list, an increase in cultured milk (+ 65%), and the addition of rye bread and tortilla bread which were not present in the baseline menu. These modifications were deemed necessary by the meal planner to compose an acceptable menu and resulted in an average reduction of 40.2% in GHGE and a 14% reduced cost compared to baseline (Table 2). The ARD increased to 16.2% and nutritional adequacy was affected marginally for energy (the new menu provided 97% of estimated energy requirements), iron (97% of RI), selenium (99% of RI), and zinc (98% of RI). In the new menu, six of the 40 served dishes were entirely vegetarian (i.e. did not containing any red meat/fish/poultry), compared to four during the baseline period (Additional file 1).

Some additional minor modifications were deemed necessary by the school chefs and these resulted in small changes to the GHGE reduction, cost, ARD and nutritional adequacy for each menu actually delivered (Table 2). These changes consisted mainly of one food item being replaced by a similar one (e.g. using white beans instead of red beans). On a few occasions, entire components were removed from the recipe (e.g. beans) without replacement, and on two occasions (one in School 1 and one in School 2) the school chefs replaced the entire main dish by a different one in order to prevent food waste.

**Fig. 1:**
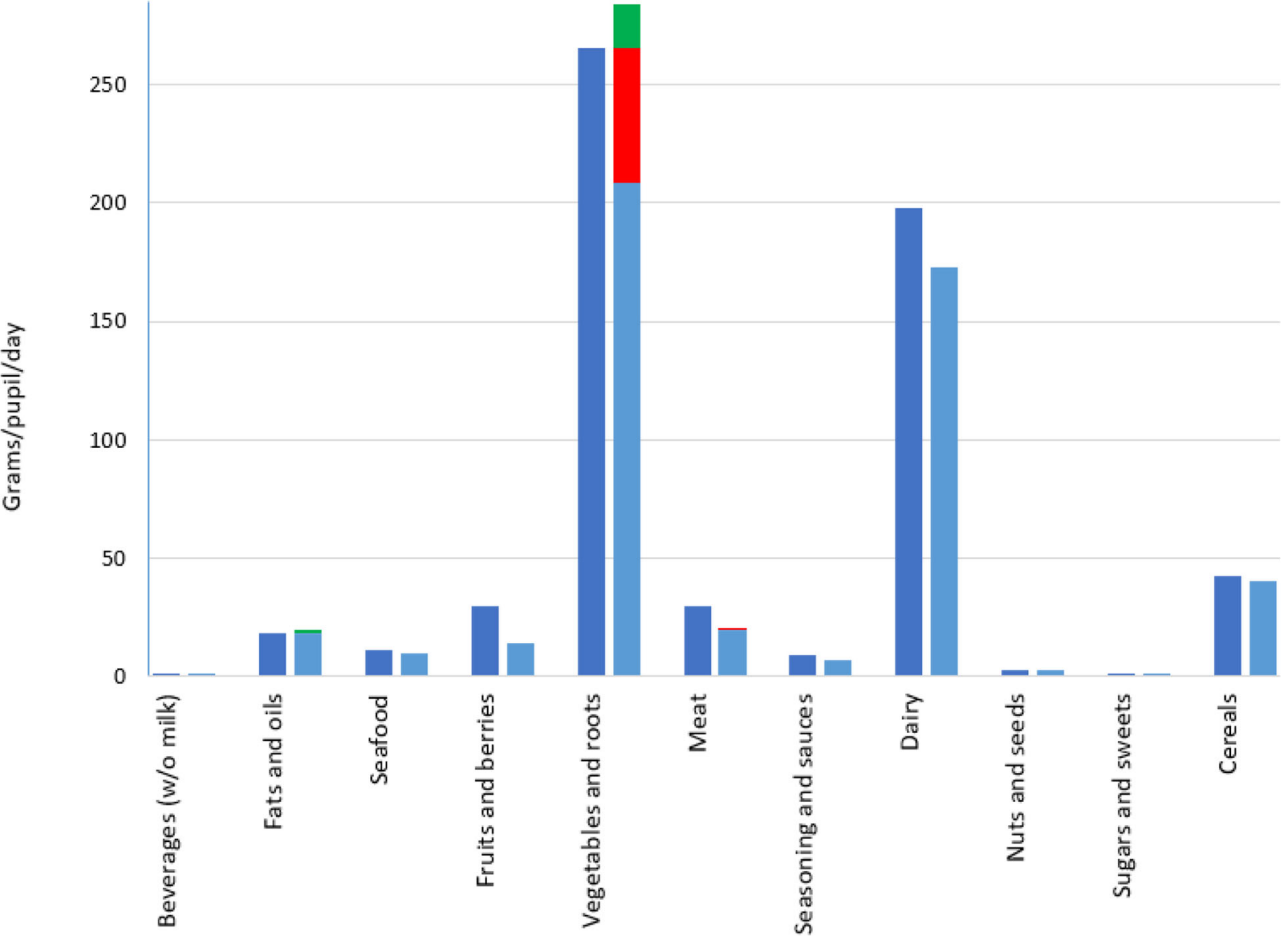
Food group quantities (g/pupil/day) before (dark blue columns) and after (light blue columns) optimization. The green part indicates the amount increased by the linear programming algorithm; The red parts indicate the amount that was removed from a food group but replaced by other foods of the same food group

Figure 1 displays the food group quantities in the baseline food list and in the optimized (isocaloric) food list. Amounts of food in all but three food groups (Beverages (without milk), Nuts and seeds, and foods categorized as Sugars and sweets) were changed by the linear programming algorithm. Two of the food groups increased in weight (Fat and oils, + 5%; Vegetables and roots, + 7%) while the remaining food groups were reduced in weight (Seafood, − 13%; Fruits and berries, − 54%; Meat, − 32%; Seasoning and sauces, − 26%; Dairy, − 13%; Cereals, − 5%).

### Food waste and consumption

Table 3 displays the mean (95% CI) daily food waste and mean (95% CI) daily food consumption during the baseline and the intervention period as well as the parameter estimates obtained from the ITS analysis. During the baseline period, approximately 15, 19 and 18% of all prepared food became either serving waste or plate waste in Schools 1, 2, and 3, respectively. During the intervention period, about 17, 19 and 21% of all prepared food became either serving waste or plate waste in Schools 1, 2, and 3.

**Table 3:** Difference in plate waste, consumption, serving waste, kitchen waste, prepared food, leftover food, and number of lunch consumers between baseline and intervention periods with 95% confidence intervals

				School 1						School 2								School 3							
		Baseline		Intervention		Parameter estimates^1^				Baseline		Intervention		Parameter estimates^1^				Baseline		Intervention		Parameter estimates^1^			
	Unit	Mean	95% CI	Mean	95% CI	*β*_*1*_	*P*	*β*_*2*_	*P*	Mean	95% CI	Mean	95% CI	*β*_*1*_	*P*	*β*_*2*_	*P*	Mean	95% CI	Mean	95% CI	*β*_*1*_	*P*	*β*_*2*_	*P*
Plate waste	kg	13.5	12.0–14.9	13.7	11.8–15.7	−1.55	0.751	0.14	0.496	17.4	15.4–19.4	19.6	17.6–21.5	0.95	0.864	−0.06	0.804	11.8	10.3–13.3	11.7	10.3–13.1	0.38	0.926	−0.06	0.722
	g/pupil	23.1	20.8–25.5	23.9	20.0–27.7	−4.09	0.648	0.34	0.370	31.5	27.7–35.4	34.9	30.9–38.8	0.15	0.989	0.03	0.948	39.1	33.1–45.2	32.2	28.4–36.0	−0.61	0.966	− 0.01	0.989
Consumption	kg	143	126–159	154	134–175	44.9	0.367	−3.3	0.124	187	151–224	185	155–215	13.49	0.882	−3.89	0.317	86	76–97	73	63–83	−0.71	0.981	−0.51	0.681
	g/pupil	245	218–272	264	231–296	69.13	0.387	−4.99	0.146	337	274–401	324	274–375	−1.1	0.994	−5.22	0.442	286	239–333	201	171–237	−35.33	0.745	0.71	0.877
Serving waste	kg	16.1	10.5–21.7	21.0	13.2–28.7	4.62	0.807	0.37	0.650	35.8	25.4–46.1	34.8	24.9–44.7	19.10	0.504	−1.20	0.327	8.7	6.6–10.8	12.8	7.9–17.6	12.64	0.233	−0.32	0.471
Kitchen waste	kg	5.4	4.1–6.8	10.0	7.9–12.0	16.40	**< 0.001**	−0.31	0.123	4.5	3.5–5.5	6.0	4.6–7.5	4.54	0.198	−0.11	0.472	3.9	2.9–4.8	3.0	2.0–4.0	−1.26	0.635	0.05	0.644
Prepared food	kg	195	177–214	209	190–228	75.46	0.134	−4.49	**0.039**	277	242–312	283	252–314	−4.10	0.963	−3.31	0.384	115	104–125	118	109–128	−2.42	0.932	0.20	0.87
Leftover food	kg	23.4	15.3–31.5	20.1	12.9–27.3	27.47	0.190	−1.69	0.067	36.4	27.6–45.1	44.0	32.7–55.3	−37.64	0.181	1.84	0.125	8.3	5.2–11.4	20.8	25.1–26.5	−15.27	0.212	1.10	**0.038**
Lunch consumers	n	582	561–603	584	559–609	11.05	0.864	−1.17	0.671	555	533–577	567	545–590	38.13	0.540	−2.66	0.317	313	285–340	368	343–394	9.03	0.897	−1.43	0.63

Bold text indicates statistically significant *p*-values (< 0.05).

^1^Parameter estimates for linear regression model with period (baseline and intervention) and interaction term time*period as independent effects, adjusted for time

*β*_*1*,_ Beta coefficient for the mean difference (baseline vs. intervention), with baseline period as the reference category

*β*_*2*,_ Beta coefficient for the slope difference (baseline vs. intervention), with baseline period as the reference category

**Fig. 2:**
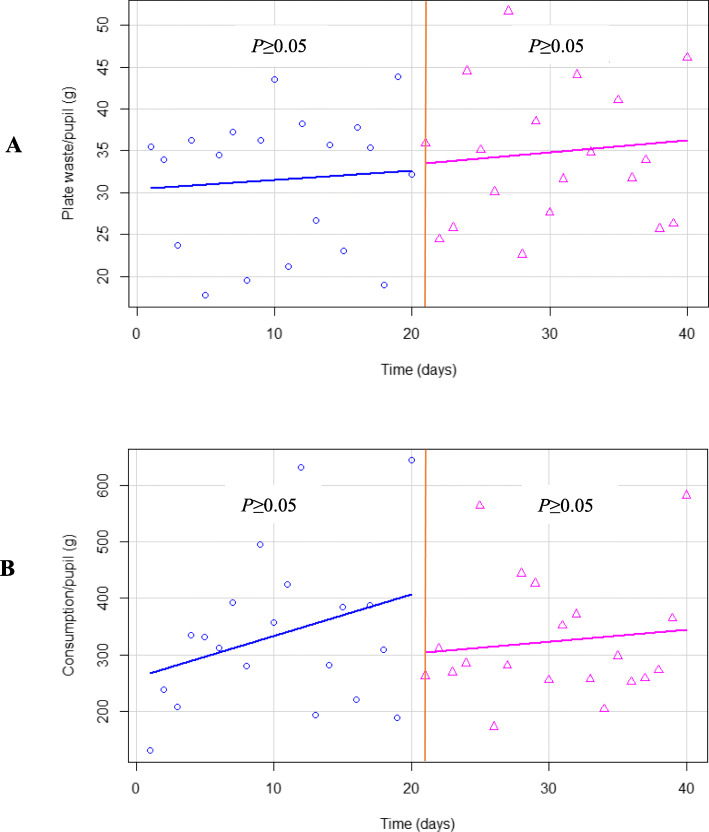
Scatterplots based on ITS analysis displaying plate waste per pupil and consumption per pupil in School 2 as an example. Panel **a** represents the daily amount of plate waste per pupil during the baseline period (measurement day 0–20, graphs to the left), and the intervention period (measurement day 21–40, graphs to the right); Panel **b** represents the daily consumption per pupil during the baseline period (measurement day 0–20, graphs to the left), and the intervention period (measurement day 21–40, graphs to the right); Vertical line represents the first day of serving the new menu; *P* ≥ 0.05 for parameter estimates (*β*_*1*_ and *β*_*2*_) from the ITS analysis

No significant differences in plate waste, serving waste or consumption were seen in any of the schools. The daily amount of plate waste (g/pupil) and consumption (g/pupil) during the baseline and intervention period are illustrated in Fig. 2 (using School 2 as an example) and in Additional file 4 (Schools 1 and 3). As evident from Fig. 2, there was a large day-to-day variation in plate waste and consumption, indicating that some dishes were more/less acceptable to pupils than others, something that also was seen in schools 1 and 3 (Additional file 4). Regarding other measurements, only the mean amount of kitchen waste in School 1 was significantly higher during the intervention as compared to baseline (*β*_*1*_ = 16.40, *p* < 0.001). Furthermore, a slope-difference between baseline and intervention was seen for the amount of prepared food in School 1 (*β*_*2*_ = − 4.49, *p* = 0.039) and for the amount of leftover food in School 3 (*β*_*2*_ = 1.10, *p* = 0.038).

### Satisfaction with school meals

During the baseline period, 86, 85, and 78% of the grade 5 and grade 8 pupils in Schools 1, 2, and 3, respectively, completed the online questionnaire (Table 4). During the last week of the intervention period, 85, 73, and 98% of grade 5 and grade 8 pupils in Schools 1, 2, and 3, respectively, completed the questionnaire (Table 4). There were no statistically significant differences in school meal satisfaction between baseline and the intervention period (Table 4).

**Table 4:** School lunch satisfaction during baseline and intervention period amongst pupils in grades 5 and 8

	All schools		p*	School 1		p*	School 2		p*	School 3		p*
	(*n* = 324^a^)			(*n* = 143^a^)			(*n* = 117^a^)			(*n* = 64^a^)		
	Baseline	Intervention		Baseline	Intervention		Baseline	Intervention		Baseline	Intervention	
	(*n* = 272^b^)	(*n* = 270^b^)		(*n* = 123^b^)	(*n* = 122^b^)		(*n* = 99^b^)	(*n* = 85^b^)		(*n* = 50^b^)	(*n* = 63^b^)	
	%	%		%	%		%	%		%	%	
Oveall rating			0.856			0.518			0.922			0.689
Very good/good	36	34		37	30		35	30		36	42	
Less good	34	35		34	38		33	38		38	31	
Bad	30	31		29	32		32	32		26	27	
Food tastes good			0.768			0.653			0.596			0.472
Always/almost always	29	27		27	24		33	28		26	34	
Rarely/never	71	73		73	76		67	72		74	66	
Frequency of eating			0.929		0.9				0.439			0.829
≤ 3 times/week	45	46		50	48		40	47		45	41	
4–5 times/week	55	54		50	52		60	53		55	59	
Satiety after lunch			0.929			0.855			0.315			0.560
Always/almost always	44	43		39	41		51	42		40	47	
Rarely/never	56	57		61	59		49	58		60	53	
Frequency of leaving plate waste			0.312			1.000			0.177			0.582
Always/almost always	34	39		33	33		36	47		32	39	
Rarely/never	66	61		67	67		64	53		68	61	

^a^Number of pupils in grades 5 and 8

^b^Number of pupils in grades 5 and 8 that answered the online questionnaire

^*^Tested by means of Pearson’s chi-squared test

## Discussion

### Main findings

The results of this study show that linear programming in combination with skilled meal planning is a useful approach in achieving a new school lunch menu that is 40% lower in GHGE, nutritionally adequate, similar in terms of composition, less costly than the original, and acceptable to pupils. We showed that the optimized school lunch menu could consequently be implemented in primary school settings with no undesirable impacts on food waste, consumption, and pupils’ school meal satisfaction. Our findings suggest that the adoption of this strategy for designing a new and more sustainable school lunch menu is applicable and acceptable in practice, thus confirming our original hypothesis.

### Interpretation

There are plenty of case studies but very little rigorous evaluation on the effects of introducing more sustainable school meals or other public meals in practice. For example, large-scale initiatives to reduce the climate impact from German school meals have been performed by offering more vegetarian options [39], yet these initiatives have not been evaluated scientifically. In Spain, goal programming (similar to linear programming but with several objective functions optimized simultaneously) has been applied to design a healthy, climate friendly, one-month lunch plan for elementary school pupils [23]. This approach resulted in GHGE-reductions of 13–24% and cost reductions of 10–15% while meeting most nutrient requirements, but the menu was not tested in practice. The optimization was made on single dishes rather than a four-week food list, which may explain the considerably smaller reduction in GHGE as compared to our study. In Finland, the introduction of a weekly compulsory vegetarian day was found to result in reduced school lunch participation, less consumption, and increased plate waste [40]. In contrast, we found that the new menu neither significantly increased the plate waste nor reduced the food consumed by pupils over a four-week period. This could be a result of the efforts we made to minimize the deviation from the baseline food supply, which was not done by any of the aforementioned initiatives [23, 39, 40]. Neither of the mentioned initiatives [23, 39, 40] implemented any specific measures or attempts to certify meal acceptability. Other researchers have also emphasized the importance of considering cultural acceptability in the modelling of climate friendlier diets [17, 20, 24, 41–44]. These researchers have addressed cultural acceptability of their optimized diet solutions by minimizing the deviation from current dietary patterns, as in the present study, while simultaneously applying nutritional and cost constraints, as well as specific targets for reductions in GHGE. By doing this, they were all able to achieve a reduction of GHGE by around 40% with relatively modest deviations from current consumption patterns. Applying linear programming to minimize the deviation from the baseline supply of individual food items thus seems to be a promising method to attain optimal solutions that are likely to be culturally acceptable while also meeting environmental, nutritional, and cost constraints [22]. This approach could thus be used by future interventions that aim to promote the adoption of more sustainable school meals.

The approach used in this study required the help of an experienced and creative meal planner who had the skills to design new menus from the optimized food list. As shown previously [24], increasing the number of constraints in the model, by e.g. requiring certain food item ratios to be kept constant, ensures that the optimized food supply is kept as similar as possible to baseline, but decreases the potential for reducing GHGE. Compromises between GHGE reduction and achieving a high similarity to current dietary patterns are needed, and as previously suggested, these types of trade-offs need to be made in collaboration with meal planners and providers [18, 24]. An alternative could be to gradually reduce GHGE over time to avoid drastic changes. Other dimensions of diet sustainability may also be conflicting. For example, a study on meals served in French primary schools showed that the nutritional quality of computer-simulated series of meals deteriorated when protein dishes were reduced or when meat−/fish-based dishes were replaced by vegetarian dishes [45]. These findings support research showing that nutritional adequacy can be compromised when animal products in diets are replaced with plant-based foods without properly considering nutritional aspects [46]. However, this problem can be solved with linear optimization. In our study, the loss of nutrients such as iron from the reduction of red meat was compensated by nutrients from more climate-friendly foods such as lentils which increased in amount to meet constraints on both reduced GHGE and nutritional adequacy. Choosing an approach (such as linear programing) that is able to balance these dynamics thus seems to be important for finding the optimal trade-offs between environmental and health priorities of diet sustainability [47].

Our results show that around 20% of the prepared food in all schools was wasted during both the baseline and the intervention period. The results from the questionnaire on school meal satisfaction further revealed a relatively low satisfaction with school meals throughout the study period. These findings align well with previous results on the Swedish school meal context showing that especially older pupils (grade 5 and above), who are allowed to leave the school grounds during the school day, tend to skip the school lunch on a regular basis [48, 49]. Furthermore, recent national data have shown that the lunch intake of pupils in grades 5 and 8 does not cover the required 30% of the daily energy and nutrient requirements and that around a third of the pupils omit at least one school lunch a week [12]. This points to the need for qualitative research to better understand how to plan school meals so that they can become both nutritionally adequate but also more acceptable to pupils. Qualitative research could provide important information regarding the challenges related to children’s attitudes towards the school lunch or to the school meal environment, which might negatively impact school lunch uptake. Involving pupils in the planning of new meals could also be a promising strategy to increase satisfaction and reduce food waste.

In this first real-world test of optimized menus, using an uncontrolled pre-post design, we decided to keep the information to pupils about the menu change to a minimum since we wanted to evaluate the effect of only changing the meals. There is evidence to suggest that interventions to improve children’s diets in schools are more likely to be effective when they combine several components such as education and environmental changes simultaneously [50, 51] and this should be explored in future studies. Findings from a school-based experiment in Finland showed that pupils’ uptake of a free daily vegetarian dish increased only when an educational campaign to increase awareness around sustainable eating was introduced [52]. Therefore, future studies should test if pupils’ eating habits and attitudes towards a more plant-based diet are influenced by the inclusion of pedagogical components. By incorporating classroom activities focusing on sustainable development, an increased understanding of the relationship between food systems, health and the environment could result, which may translate to positive attitudes towards eating climate-friendly lunches. Such a multi-component approach is suggested to be effective as it combines both intuitive and reflective cognitive processes [53]. Swedish schools already have learning goals for sustainable consumption and development, and so the more active involvement of teachers and pupils in the process of adopting healthier and more sustainable school meals could be justified.

### Strengths and limitations

This unique study has tested a theoretically modeled diet in a real-world setting, providing a steppingstone towards promoting sustainable dietary habits in practice. Considering the contextual differences both within and between countries and regions, the applied approach is useful as it offers the flexibility to modify the objective function and/or constraints so that the priorities and needs of the user can be accommodated. This approach could therefore be transferred to many other types of meal services.

Another strength of the present paper is the ITS analysis, enabling a detailed analysis of the effect of the intervention on food waste and consumption in each school. For example, in School 1, the ITS-analyses did not show a mean-difference in the amount of prepared food but revealed a significant slope-difference in this variable between baseline and the intervention period. This could be explained by a change in the amount of prepared food during a specific time-sequence of the intervention period, which significantly altered the slope of the regression line but without significantly affecting the mean-amount of prepared food. The reason for this was investigated by interviewing the pupils and kitchen staff and results are currently being synthesized for publication in a forthcoming paper. Simple before-after comparisons (e.g. t-tests) would be less likely to provide these insights [25].

There are also some limitations which need to be considered. This study was performed in only three Swedish schools, limiting its generalizability. Furthermore, the study’s pre-post design, without a control group, limits the interpretation of results since the observed changes could also be a result of underlying secular trends in society. However, the study period was relatively short (10 weeks in total) and therefore it is unlikely that secular trends such as increased food prices or seasonal variation had any impact on the results. Additionally, the change of menu was limited to a four-week period and this might not be considered long enough to observe significant acceptance on the modified menu in a longer perspective. However, overall acceptance would if anything be expected to increase rather than decrease over a longer period of time as increased exposure is a factor known to positively influence acceptance of new foods [54].

The applied models did not take the bioavailability of important nutrients into account. For example, red meat (reduced by the linear programming algorithm) has a high bioavailability of iron compared to tubers or pulses (increased by the linear programming algorithm) [55]. This may negatively impact the micronutrient status in vulnerable populations [55]. Future modelling studies could benefit from taking bioavailability of critical micronutrients such as iron into consideration [56].

Many new foods with low GHGE as well as products fortified with nutrients traditionally lacking in vegetarian diets, are emerging on the market [57, 58]. Including these foods in the optimization of new menus could provide certain benefits but potentially also result in greater dietary changes for pupils, which could lead to reduced acceptability [42, 59]. However, future studies should explore the effects of also including such foods in the modelling as they offer untapped opportunities to further reduce the climate impact of meals.

## Conclusions

This is the first study to explore the practical applicability and acceptability of implementing more sustainable school meals using linear programming. Our findings demonstrate that this approach can be implemented in schools, and therefore potentially also in other settings that wish to procure and serve more climate friendly meals. Ideally, future research would involve larger-scale and longer-term interventions and also include educational components, to understand the full potential of these opportunities. Such knowledge may play a role in contributing to lower climate impact of food and ultimately to achieving the United Nations Sustainable Development Goals and mitigating climate change.

## Supplementary information

**Additional file 1: Supplementary Table 1.** Dishes in the four-week baseline and new menu plans, respectively. The second dish at baseline is always vegetarian while in the new menu the first dish is. The meals are served with a salad buffet, bread and butter or margarine.

**Additional file 2: Supplementary Table 2.** Nutrient constraints applied during all linear optimization procedures.

**Additional file 3: Supplementary Fig. 1.** Questionnaire about satisfaction with school meals.

**Additional file 4: Supplementary Fig. 2.** Scatterplots displaying plate waste per pupil and consumption per pupil in Schools 1 and 3.

**Additional file 5: Supplementary Table 3.** TIDieR Checklist for intervention components.

## Data Availability

The datasets generated and/or analysed during the current study are available in the Mendeley repository, https://data.mendeley.com/datasets/8tgd6jbr67/draft?a=6128af00-bc1c-434c-a970-c5ff87377485

## Abbreviations

ARD: Average relative deviation
CBC: Coin-or branch and cut
CI: Confidence interval
CO_2_eq: Carbon dioxide equivalents
EUR: Euro
GHGE: Greenhouse gas emissions
ITS: Interrupted time series
LP: Linear programming
RI: Recommended intake
RISE: Research Institutes of Sweden
SCT: Social cognitive theory
SEK: Swedish Krona
TRD: Total relative deviation
