# Supplementary material for: Sustainable and acceptable school meals through optimization analysis: an intervention study
Source: Nutr J. 2020 Jun 24;19:61. doi: 10.1186/s12937-020-00579-z (PMC7315552; doi:10.1186/s12937-020-00579-z)
Supplement: Supplementary file 1 — Additional file 1: Supplementary Table 1. Dishes in the four-week baseline and new menu plans, respectively. The second dish at baseline is always vegetarian while in the new menu the first dish is. The meals are served with a salad buffet, bread and butter or margarine. [file 12937_2020_579_MOESM1_ESM.pdf]

Supplementary Table 1. Dishes in the four-week baseline and new menu plans, respectively. The second dish at baseline is always vegetarian while in the new menu the first dish is. The meals are served with a salad buffet, bread and butter or margarine.

	Baseline	New
	Week 1	
Mon	Smoked pork with Greek tomato sauce and grated cheese, bulgur	Broccoli gratin, bulgur
	Broccoli gratin, bulgur	Bulgur with smoked pork sauce
Tue	Chicken with green pepper and tarragon sauce, rice	Quorn fillet with tarragon and pepper sauce, pommes chateau
	Quorn filet with green pepper and tarragon sauce, rice	Chicken fillet with tarragon and pepper sauce, pommes chateau
Wed	Breaded fish patties with remoulade sauce, boiled potato	Vegetable patty, cold sauce, boiled potatoes
	Vegetable patty, cold sauce, boiled potato	Breaded fish patties with herb sauce, boiled potatoes
Thu	Creamy potato soup, bread, cold cuts, fruit	Carrot soup, bread with carrot pesto, fruit
	Carrot soup, bread, cold cuts, fruit	Potato and leek soup, bread with cheese, fruit
Fri	Beef lasagne	Vegetarian moussaka
	Vegetarian lasagne	Middle Eastern stew with garlic yogurt, bulgur
	Week 2	
Mon	Hen stew with curry sauce, rice	Chickpea ratatouille, bulgur
	Ratatouille, bulgur	Hen with curry sauce, boiled potatoes
Tue	Pasta gratin with feta cheese and spinach	Colorful pasta wok with chili yogurt
	Root vegetable hash with basil sauce	Mediterranean pasta with pea pesto
Wed	Oven baked fish with gremolata, boiled potatoes	Thai chickpea stew, rice
	Thai chickpea stew with red curry, potatoes	Oven baked fish with gremolata, boiled potatoes
Thu	Baked sausage, potato	Root vegetable patties with bulgur salad and cottage cheese
	Vegetarian burritos	Root vegetable stew with sausage, boiled potatoes
Fri	Middle Eastern meat stew, yogurt, bulgur	Red lasagne with thyme and cottage cheese
	Vegetarian moussaka	Lasagne
	Week 4	
Mon	Pasta Carbonara	Pasta with spinach and garlic sauce
	Pasta with spinach and garlic sauce	Pasta Carbonara
Tue	Coconut chicken with sweet chili sauce, yogurt, rice	Noodle wok with soy strips and sweet chili sauce
	Noodle stir-fry with soy strips and sweet chili sauce	Coconut chicken with sweet chili sauce, rice
Wed	Boiled fish with parsley sauce, boiled potato	Couscous hash, red beans
	Couscous hash, garlic sauce	Poached fish with parsley sauce, broccoli, lemon hummus, potato
Thu	Carrot soup with lentils, bread, cold cuts, fruit	Orange and carrot soup, bread, cold cuts and fruit
	Vegetarian goulash soup, bread, cold cuts, fruit	Vegetarian goulash soup, bread, cold cuts, fruit
Fri	Meatloaf with sauce, boiled potatoes	Chickpea patties with roasted root vegetables and lentil cream
	Chickpea patties with oven baked vegetables, potatoes	Beef and bean casserole with cream sauce, boiled potatoes
	Week 4	
Mon	Mediterranean gratin with pasta	Root vegetable hash with curry sauce
	Pasta with broccoli, spinach and cheese sauce	Pasta gratin with feta cheese and spinach
Tue	Chicken nuggets with sweet and sour sauce, rice	Burritos with vegetable pisto
	Mung bean nuggets, with sweet and sour sauce, rice	Chicken nuggets with sweet and sour sauce, whole wheat
Wed	Fish patties with cold sauce, boiled potatoes	Carrot patty, cold sauce, oven roasted potatoes
	Carrot patties, cold sauce, boiled potatoes	Fish patties with chili, with cold sauce, green peas, boiled potatoes
Thu	Pork gratin, wheatberries	Vegetarian pasta gratin
	Vegetarian pasta gratin	Potato gratin with ham and pickled carrots
Fri	Indian meat stew, rice	Indian lentil dhal, fresh bread and fruit
	Indian lentil dhal	Mungbean nuggets, with sweet and sour sauce, wheatberries
