# Supplementary material for: Sustainable and acceptable school meals through optimization analysis: an intervention study
Source: Nutr J. 2020 Jun 24;19:61. doi: 10.1186/s12937-020-00579-z (PMC7315552; doi:10.1186/s12937-020-00579-z)
Supplement: Supplementary file 2 — Additional file 2: Supplementary Table 2. Nutrient constraints applied during all linear optimization procedures. [file 12937_2020_579_MOESM2_ESM.pdf]

**Supplementary Table 2.** Nutrient constraints applied during all linear optimization procedures.

| Upper and/or lower limits that the optimized food supply had to comply with | | |
|---|---|---|
| | Lower limit | Upper limit |
| Energy (kcal)[a] | 674 | 674 |
| Carbohydrates (%E) | 45 | 60 |
| Fat (%E) | 22 | 40 |
| Protein (%E) | 10 | 20 |
| Fiber (%E)[c] | 2 | — |
| Saturated fatty acids (%E) | — | 10 |
| Mono/disaccharides (%E) | | 10 |
| Mono unsaturated fatty acids (%E) | 10 | 20 |
| Poly unsaturated fatty acids (%E) | 5 | 10 |
| Vitamin A (µg)[b] | 188 | — |
| Vitamin D (µg)[b] | 3.1 | — |
| Vitamin E (mg)[b] | 2.2 | — |
| Thiamine (mg)[b] | 0.3 | — |
| Riboflavin (mg)[b] | 0.4 | — |
| Vitamin C (mg)[b] | 16.0 | — |
| Niacin (mg)[b] | 4.6 | — |
| Vitamin B6 (mg)[b] | 0.4 | — |
| Vitamin B12 (µg)[b] | 0.6 | — |
| Folate (µg)[b] | 62.7 | — |
| Phosphor (mg)[b] | 199 | — |
| Iodine (µg)[b] | 43.2 | — |
| Iron (mg)[b] | 3.4 | — |
| Calcium (mg)[b] | 282 | — |
| Potassium (mg)[b] | 971 | — |
| Magnesium (mg)[b] | 87.7 | — |
| Salt (g)[c] | — | 3.6 |
| Selenium (µg)[b] | 12.5 | — |
| Zink (mg)[b] | 3.4 | — |
| Omega-3 fatty acids (%E)[b] | 3.3 | — |

[a]Based on 30% of daily estimated energy requirement (EER) for an average pupil, 10-12 years, both sexes.
[b]Lower limit based on 30% of daily recommended intakes (RIs) for an average pupil, 10-12 years, both sexes.
[c]Upper limit based on 30% of daily recommended intake (RIs) for an average pupil, 10-12 years, both sexes.
