# Supplementary material for: Sustainable and acceptable school meals through optimization analysis: an intervention study
Source: Nutr J. 2020 Jun 24;19:61. doi: 10.1186/s12937-020-00579-z (PMC7315552; doi:10.1186/s12937-020-00579-z)
Supplement: Supplementary file 3 — Additional file 3: Supplementary Fig. 1. Questionnaire about satisfaction with school meals. [file 12937_2020_579_MOESM3_ESM.pdf]

Skolmat SVERIGE.SE

Supplementary Figure 1. Questionnaire about satisfaction with school meals.

In what grade are you? *

- ☐ Grade 5
- ☐ Grade 8

Are you a girl or a boy?

- ☐ Girl
- ☐ Boy

How often do you usually eat lunch in the school restaurant? *

How often do you take food from the school restaurant, even if it is just a sandwich?

- ☐ Never or almost never
- ☐ 1 day a week
- ☐ 2 days a week
- ☐ 3 days a week
- ☐ 4 days a week
- ☐ 5 days a week

If you sometimes do not eat from the hot, cooked school lunch, what is most often the reason? *

Only chose one alternative.

- ☐ I always eat the school lunch!
- ☐ I am not hungry
- ☐ I do not think the food tastes good
- ☐ I do not have enough time
- ☐ I do not like the environment in the school restaurant
- ☐ My friends do not eat the school lunch
- ☐ Other

The food

Now think about how you usually feel about the school lunch. Try not only to think of today's or yesterday's lunch!

Which face seems to represent what you think about the questions below? Put a cross in the box below the correct face.

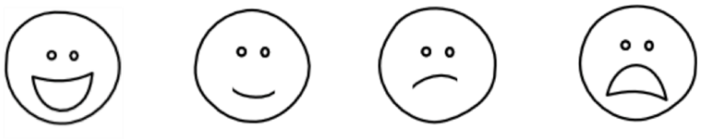

	Always	Almost always	Rarely	Never
**Does the food taste good? ***	☐	☐	☐	☐
**Do you usually feel full after eating the lunch? ***	☐	☐	☐	☐

Do you have a non-standard diet? **For example due to allergy, intolerance, religion or other. The question is voluntary to answer.**

☐ Yes
☐ No
☐ Don't know

Time

Now think about the time you usually have for eating the school lunch. Try not only to think of today's or yesterday's lunch!

	Always	Almost always	Rarely	Never
**Do you always have enough time to eat your school lunch? ***	☐	☐	☐	☐

Noise levels

Now think about how the school restaurant usually is. Try not only to think of today's or yesterday's lunch!

	Always	Almost always	Rarely	Never
	☐	☐	☐	☐

Is the noise level in the school restaurant ok/acceptable? *

The food you take but do not eat

	Always	Almost always	Rarely	Never
**How often do you leave food on the plate? ***	☐	☐	☐	☐

If you leave food on your plate, what is usually the reason for that? *

Only chose one alternative.

- ☐ I never leave food on my plate!
- ☐ I am not as hungry as I think I am
- ☐ The food does not taste as good as I think
- ☐ I take too much food as I am afraid that the food will run out
- ☐ I don't have enough time to eat everything
- ☐ I don't know why

Overall rating

Now think about the school lunch as a whole. Which face seems to represent what you think about the question below? Put a cross in the box below the correct face.

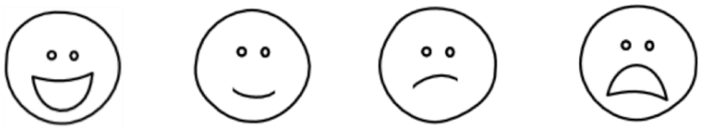

	Very good	Good	Less good	Bad	Don't know
**What overall grade do you give the school lunch at your school? ***	☐	☐	☐	☐	☐

Do you have any suggestions on how the school lunch at your school can be improved?

Please write here!
