# Supplementary material for: Sustainable and acceptable school meals through optimization analysis: an intervention study
Source: Nutr J. 2020 Jun 24;19:61. doi: 10.1186/s12937-020-00579-z (PMC7315552; doi:10.1186/s12937-020-00579-z)
Supplement: Supplementary file 4 — Additional file 4: Supplementary Fig. 2. Scatterplots displaying plate waste per pupil and consumption per pupil in Schools 1 and 3. [file 12937_2020_579_MOESM4_ESM.pdf]

Supplementary Figure 2. Scatterplots displaying plate waste per pupil and consumption per pupil in Schools 1 and 3.

A

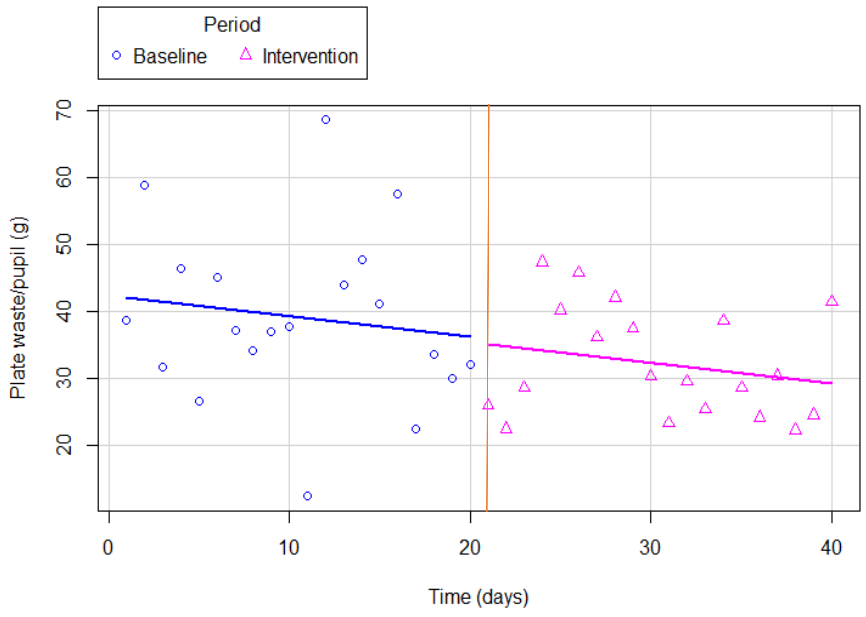

B

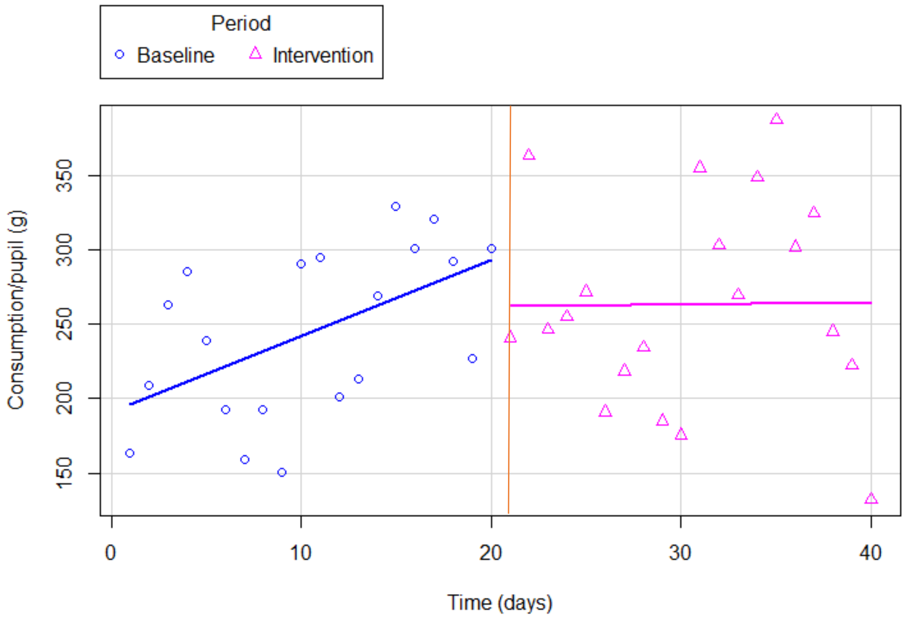

C

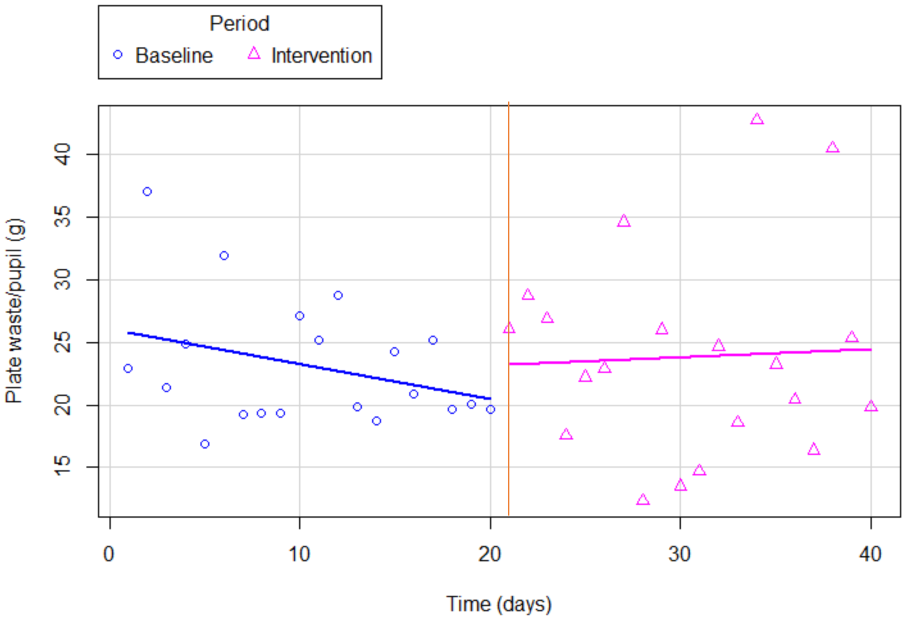

D

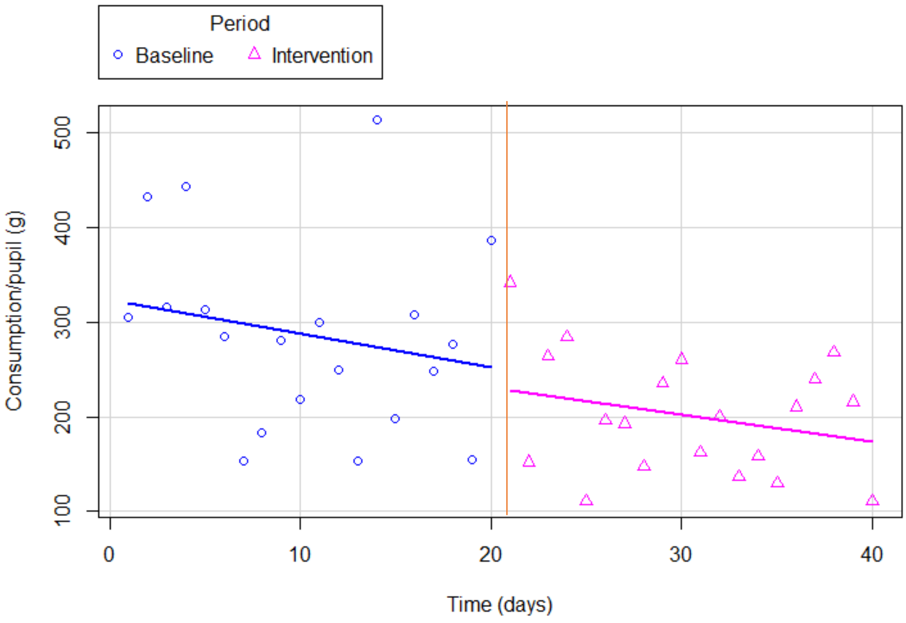

Panels **A** and **C** represent the daily amount of plate waste per pupil during the baseline period (measurement day 0-20, graphs to the left), and the intervention period (measurement day 21-40, graphs to the right) in Schools 1 and 3, respectively; Panels **B** and **D** represent the daily consumption per pupil during the baseline period (measurement day 0-20, graphs to the left), and the intervention

period (measurement day 21-40, graphs to the right) in Schools 1 and 3, respectively; Vertical line represents the first day of serving the new menu.
